# Index-Catalogue of the Library of the Surgeon-General’s Office, United States Army

**Published:** 1884-11

**Authors:** 


**Index-Catalogue of the Library of the Surgeon-General's Office, United States Army.** Authors and subjects. Vol. V. Washington: Government Printing Office. 1884.

The fifth volume of the catalogue carries on the index from FL to HE. That more than 1,000 pages are filled with titles under these alphabetical headings is an indication of the magnitude of the work which is being so successfully and perseveringly accomplished under Dr. Billings. This volume includes 15,555 author-titles, 8,069 subject-titles of separate books and pamphlets, and 34,127 titles of articles in periodicals. We earnestly hope that nothing may arise to hinder the active prosecution of this great work. When completed, it will form the most complete index to medical literature in the world.

